# Comparison of End-to-End Technique, Helicoid Technique, and Modified Helicoid Weave Repair Technique in a Rat Sciatic Nerve Model: A Pilot Study

**DOI:** 10.7759/cureus.9196

**Published:** 2020-07-15

**Authors:** Laxminarayan Bhandari, Yoram Fleissig, Robert Hoey, Jason E Beare, Christine Yarberry, Shiro Yoshida, Tsu-min Tsai

**Affiliations:** 1 Hand and Microsurgery, Christine M. Kleinert Institute, Louisville, USA; 2 Anatomical Sciences & Neurobiology, University of Louisville, Louisville, USA; 3 Medicine, Cardiovascular Innovation Institute, University of Louisville, Louisville, USA; 4 Medicine, Kentucky Spinal Cord Injury Research Center, University of Louisville, Louisville, USA; 5 Neurological Surgery, Kentucky Spinal Cord Injury Research Center, University of Louisville, Louisville, USA

**Keywords:** nerve repair, sciatic nerve injury, helicoid technique, nerve repair techniques

## Abstract

Background

The gold standard for nerve repair is end-to-end (ETE) repair. Helicoid technique (HT) has also been previously described. In this pilot study, HT was compared to ETE and a modified helicoid weave technique (MHWT). In MHWT, recipient nerve is passed through rather than around the donor nerve, allowing for greater nerve-to-nerve interaction.

Methods

Eighteen adult male Lewis rats received a 2-cm sciatic nerve transection and were divided into three groups: ETE, HT, and MHWT. Five months later, electromyography (EMG), tetanic force of contraction, and wet weight of the extensor digitorum longus muscle were recorded in both the operated and non-operated sides. Nerve biopsies were taken proximal and distal to the site of the nerve graft for histological examination.

Results

One rat died following repair surgery and three rats died during the second surgery. The mean threshold of stimulation for ETE, HT, and MHWT were 183.3 µA, 3707.5 µA, and 656.6 µA, respectively. EMG analysis revealed that latency and duration are both affected by surgical repair type and injured or uninjured conditions. Threshold ratio (injured:non-injured) revealed pilot-level significant differences between HT and both MHWT (p = 0.069) and ETE (p = 0.082). Nerve biopsy demonstrated fascicles distally in all three groups.

Conclusions

While HT and MHWT function as a nerve repair technique, they are not superior to ETE. ETE remains the gold standard for nerve repair. While mean values were in favor of ETE, no statistical significance was attained.

## Introduction

Peripheral nerve injuries are common and comprise 2-3% of all trauma cases [1]. If left untreated, the patient can be subjected to lifelong disability, pain, and an impaired quality of life [1]. Timely surgery and tensionless primary repair of clean cut ends are associated with the best outcome [2]. End-to-end (ETE) repair is the gold standard technique for nerve repair. While contemplating nerve transfers, end-to-side (ETS) repair appears a valuable alternative as it can potentially spare the donor nerve.

Another method of nerve repair known as the helicoid technique (HT) has also been proposed. HT is a modification of ETS originally described in 2002 by Yan et al. [3]. In this method, a large perineural window is created in the donor nerve. The recipient nerve is trimmed diagonally and wrapped in a helicoid fashion around the longitudinal axis of the donor nerve at a 30-degree angle. The hypothesis behind HT is two-fold. Firstly, HT enables larger area for nerve-to-nerve interaction, facilitating a large proportion of donor nerve sprouting into the recipient nerve. Secondly, the recipient nerve can interact with a larger topographical area of the donor nerve with different nerve fibers on the circumferential aspects by helicoid attachment. Such an arrangement can potentially decrease the likelihood of missing the intended donor fascicle. Yan et al. used this technique first in a rat tibial-peroneal nerve model and found a greater amplitude of conduction, tetanic force of contraction (TFC), and weight of the target muscle, as well as higher nerve fiber count distal to repair compared to ETS [3]. This technique was further tested in a rat phrenic-musculocutaneous nerve (MCN) model and was shown to be superior to ETE in terms of the amplitude of conduction, the TFC, and weight of the target muscle, as well as nerve density distal to the repair [4]. Similarly, the authors showed a superior outcome of the vagus to musculocutaneous transfer by HT over phrenic to musculocutaneous transfer by ETE in terms of the amplitude of conduction, the TFC, and weight of the target muscle, as well as higher nerve fiber count distal to repair [5]. However, these reports have not been reproduced elsewhere, and therefore there is a need to validate these findings to see if these results are reproducible.

The aim of this study was an attempt to reproduce the results of HT and compare it to ETE. In addition, we proposed a modification of HT called the modified helicoid weave technique (MHWT), wherein the recipient nerve was passed through, instead of around, the donor nerve. We hypothesized that this technique would provide an increased cross-sectional area for nerve-to-nerve contact, which would yield higher axonal crossover. Also, there will be multiple perineural windows with controlled perineural injury, each potentially a site for spontaneous axonal sprouting [6]. In this study, we compared all three techniques, HT, ETE, and MHWT, for the amplitude of conduction, the TFC, and weight of the target muscle, as well as the presence of nerve fiber distal to repair.

## Materials and methods

**Figure 1:**
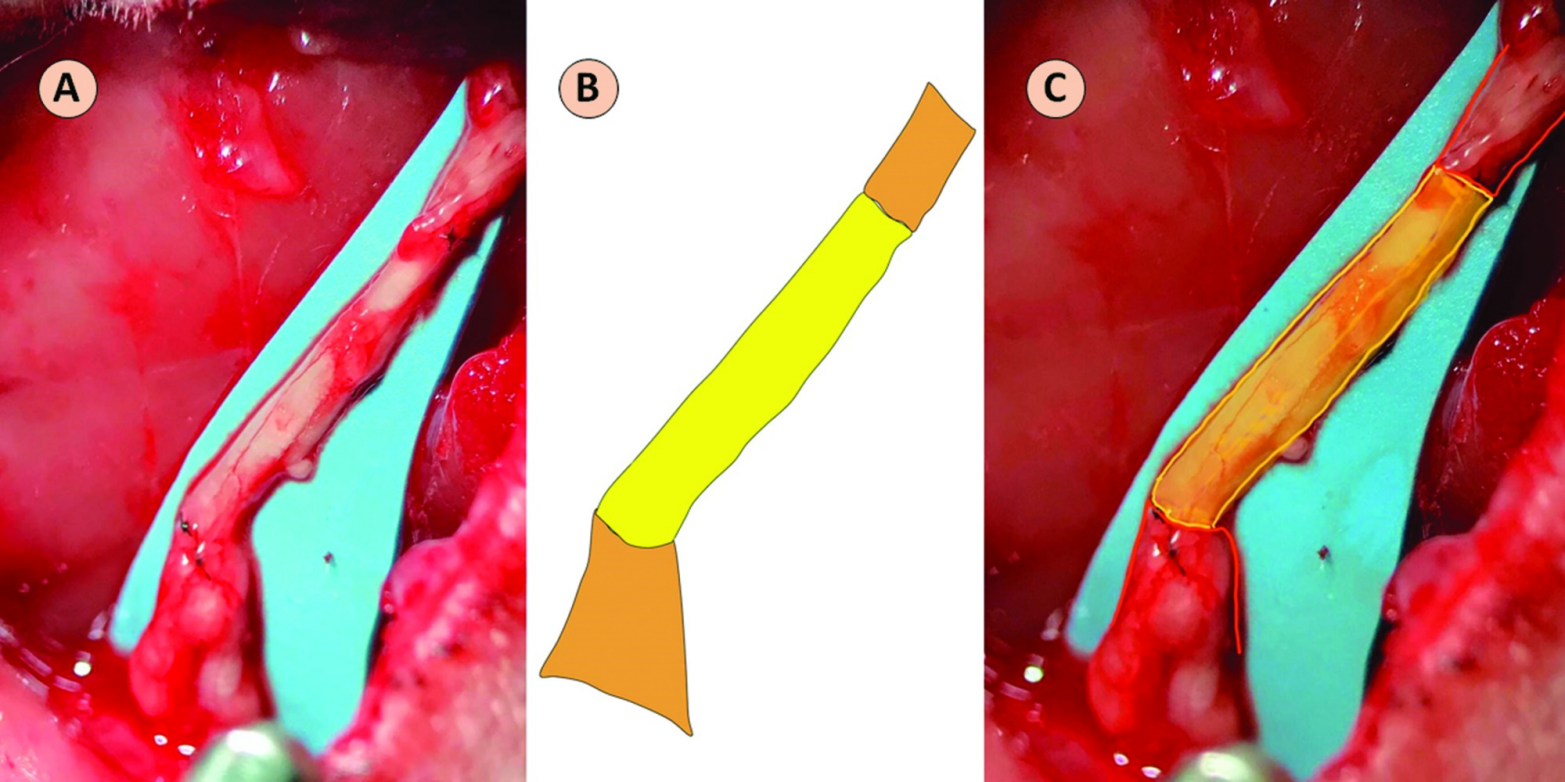
End-to-End Technique A 2-cm portion of rat sciatic nerve that was transected and then repaired back in its position. (A) Intraoperative image. (B) Schematic diagram showing the sciatic nerve (orange) and the graft (yellow). (C) Superimposed schematic on the intraoperative image.

The study was conducted utilizing a sciatic nerve model in 18 adult male Lewis rats (275-325 g). Procedures were performed in accordance with the University of Louisville Institutional Animal Care and Use Committee (IACUC protocol #17009) and the NIH Guide for the Care and Use of Laboratory Animals [7]. Power analysis based on previous studies indicated six rats to be allotted to each group [3-5]. Animals were anesthetized with 2.5% inhaled isoflurane followed by intraperitoneal injection of ketamine/xylazine. A 2-cm gap was created in the sciatic nerve. As reported by Kaplan et al., the critical nerve gap, defined as a nerve gap over which no spontaneous recovery will occur without some form of nerve grafting or bridging, is 1.5 cm for rat sciatic nerve [8]. For the ETE group, the excised 2-cm nerve was placed back and sutured in place with 10-0 nylon (Figure 1).

In the HT and MWHT groups, a 3.4-cm nerve graft was needed to bridge the 2-cm nerve gap since there is about 0.7 mm overlap on both ends. This nerve graft was taken from a separate donor Lewis rat. We did not take the opposite sciatic nerve as graft because loss of both sciatic nerves would have caused severe morbidity and suffering to the animal. Since Lewis rats are isogenetic, there is no risk of rejection of the graft.

**Figure 2:**
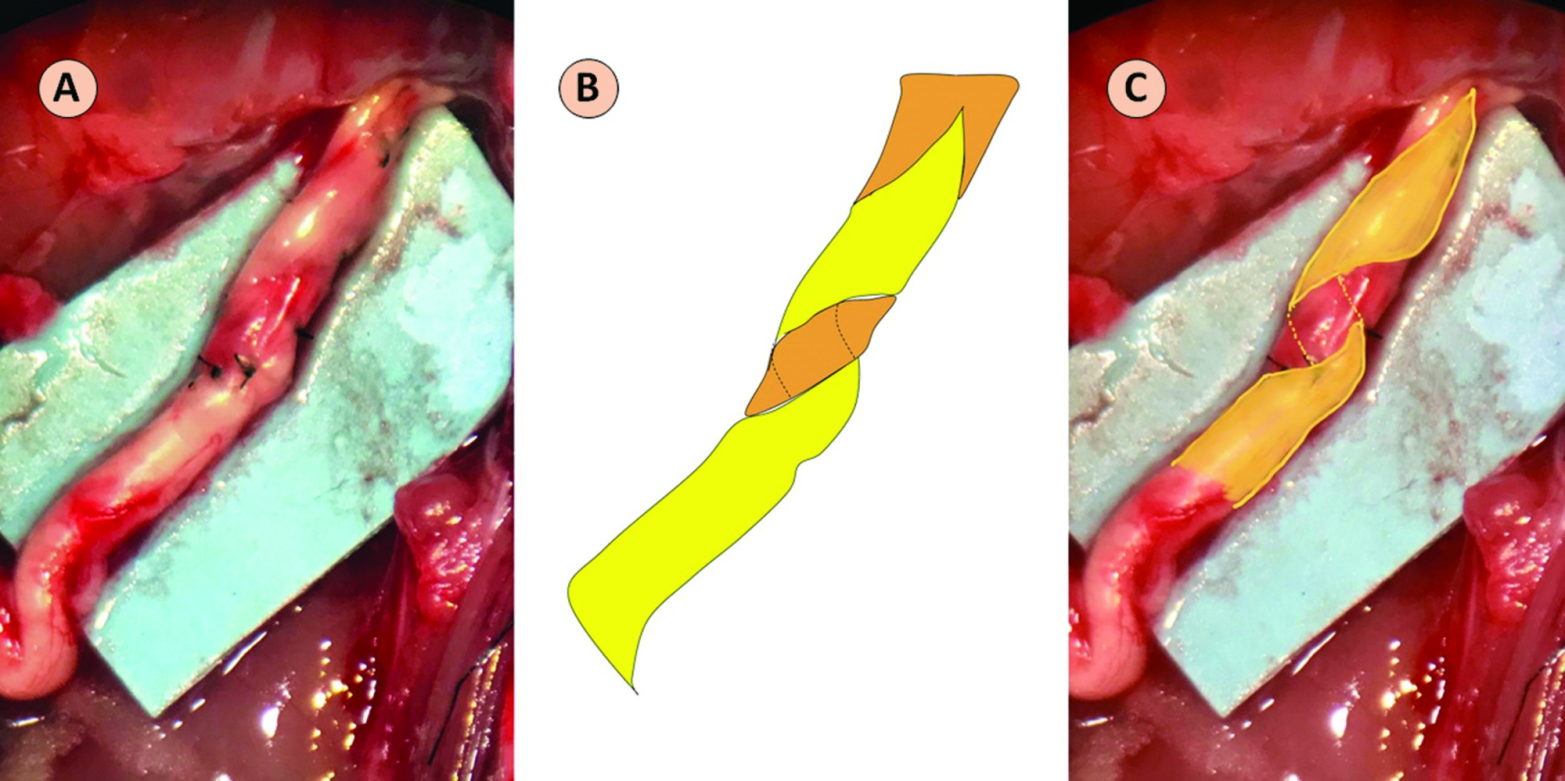
Helicoid Technique The nerve graft is wrapped around the sciatic nerve. (A) Intraoperative image. (B) Schematic diagram showing the sciatic nerve (orange) and the graft (yellow). (C) Superimposed schematic on the intraoperative image.

In the HT group, the nerve graft was cut obliquely. A large circumferential perineural window was created on the sciatic nerve. The graft was wrapped around the sciatic nerve at both the proximal and distal ends (Figure 2).

**Figure 3:**
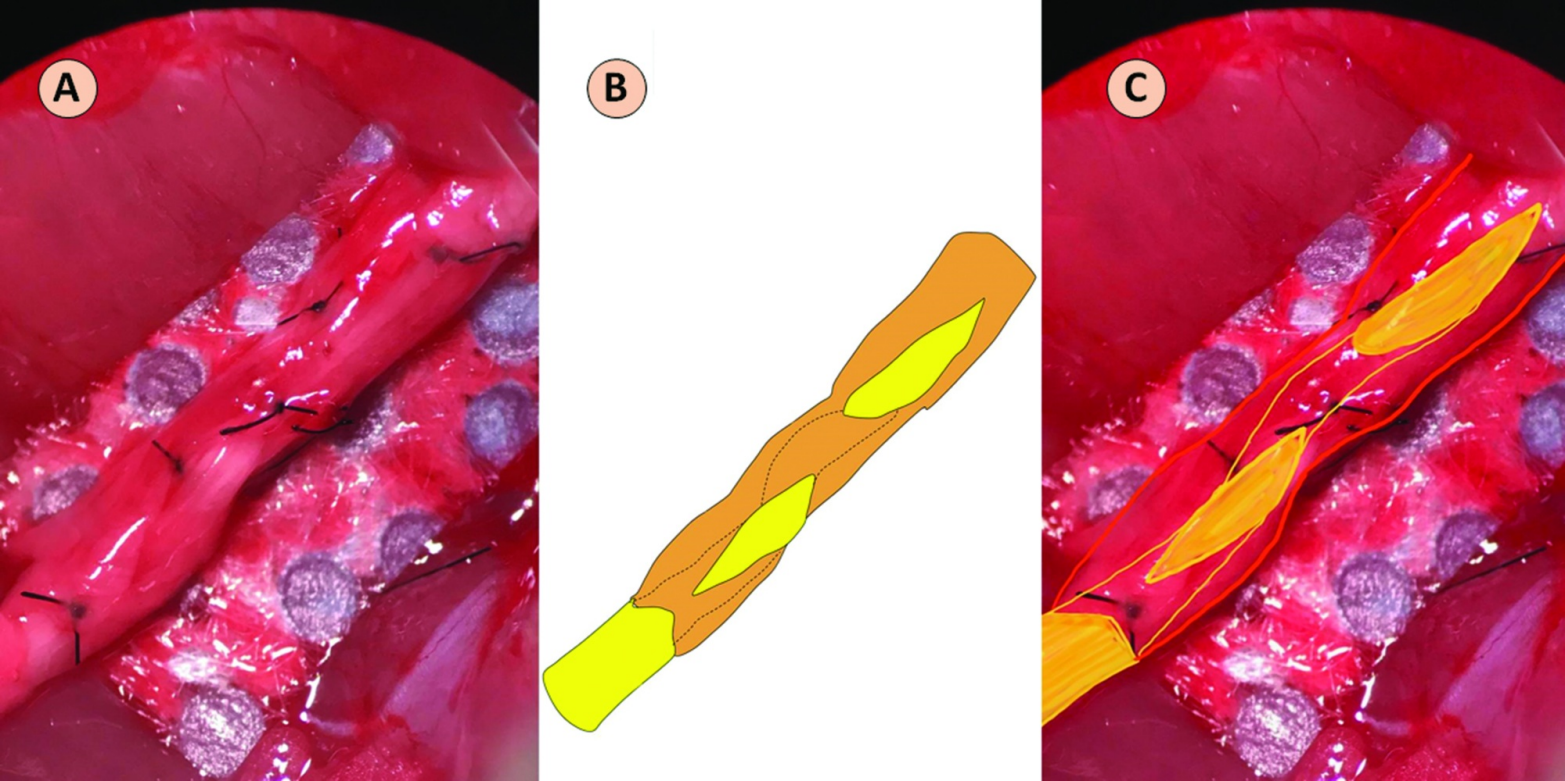
Modified Helicoid Weave Technique (A) Intraoperative image. (B) Schematic diagram showing the sciatic nerve (orange) and the graft (yellow). One end of the nerve isograft (yellow) was sutured ETS to the proximal sciatic nerve (right-hand side). The graft was then passed through the sciatic nerve (orange) two times. The proximal end of the cut sciatic nerve was repaired ETS to the graft (left-hand side). (C) Superimposed schematic on the intraoperative image. ETS, end-to-side

In the MWHT group, one end of the nerve isograft was sutured ETS to the donor nerve, in this case the sciatic nerve. The graft was then passed through the sciatic nerve two times. At each pass, the part of the graft being inside the sciatic nerve was circumferentially denuded of its epineurium to allow nerve-to-nerve interaction. The proximal end of the cut sciatic nerve was repaired ETS to the graft (Figure 3). A similar repair was performed between the nerve graft and the distal end of the sciatic nerve.

**Figure 4:**
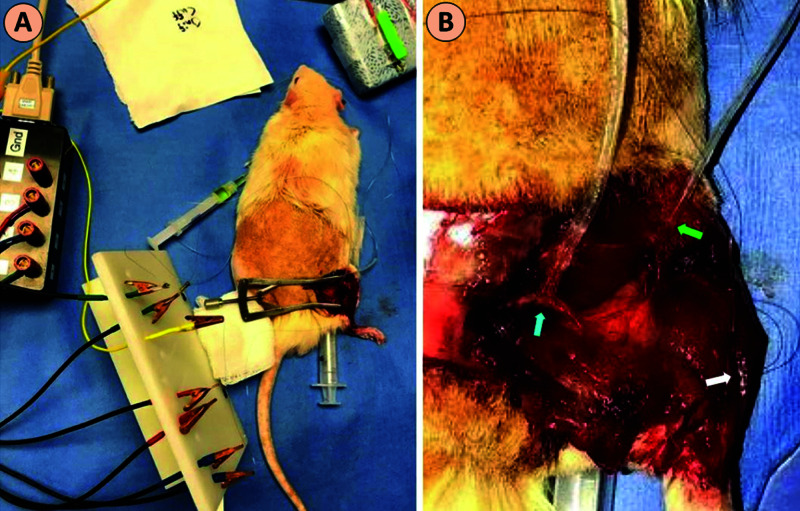
Electrophysiological Testing (A) Setup of electrophysiological testing. (B) A stimulating electrode cuff was placed on the sciatic nerve (blue arrow), the recording electrode cuff was placed on the peroneal nerve (green arrow), and the EMG recording probe was placed in the EDL muscle (white arrow). EMG, electromyography; EDL, extensor digitorum longus

After a five-month interval, a second surgery was performed through the same surgical approach. Nerves were exposed and cuffed using in-house fabricated silicone cuffs which utilized two wires for stimulation or recording of neural tissue. Fine-wire electromyography (EMG) electrodes were placed into the body of the extensor digitorum longus (EDL) muscle, approximately 1 mm apart from each other, with a ground electrode placed in the tail (Figure 4).

Nerve conduction

Stimulation was applied to the sciatic trunk first by finding a threshold and then applying increasing stimulation intensities (500 µA, 1, 2, 4, 8, and 15 mA; 1 Hz, 0.01 ms duration). Bilateral recordings were done to compensate for anesthetic and temperature variations. Responses were recorded for both the uninjured and injured nerves in each animal. Latency to response and duration of M-wave were quantified.

Threshold of stimulation

The stimulation was started with 50 µA current and gradually increased until the EMG recording first became visible. The current in the stimulating electrode when EMG recording began to appear were noted as the threshold of stimulation.

Tetanic force of contraction

**Figure 5:**
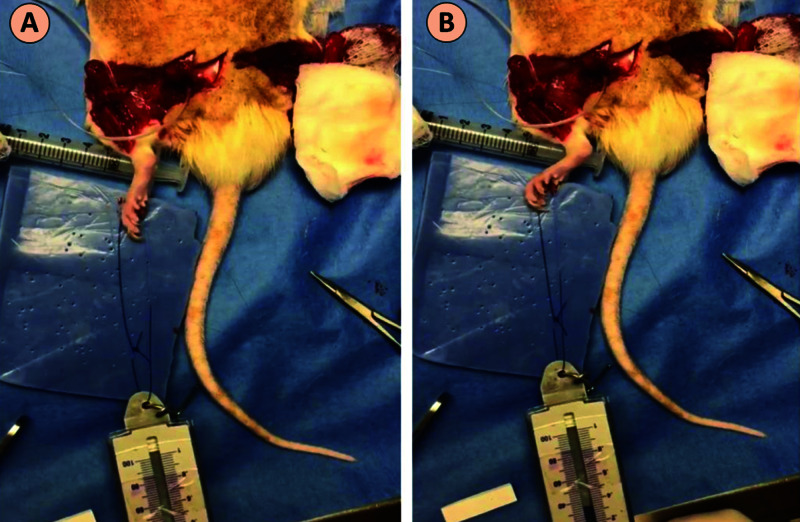
Tetanic Force of Contraction (A) Setup for the measurement of tetanic force of contraction. (B) Peroneal nerve probes were stimulated at 15 mA at 60 Hz and contraction force was measured by a spring scale.

Peroneal nerve probes were reversed and stimulated at 15 mA at 60 Hz. This produced powerful dorsiflexion of the ankle. The feet were connected to a spring balance to measure the strength of ankle dorsiflexion (Figure 5).

Wet weight of EDL

**Figure 6:**
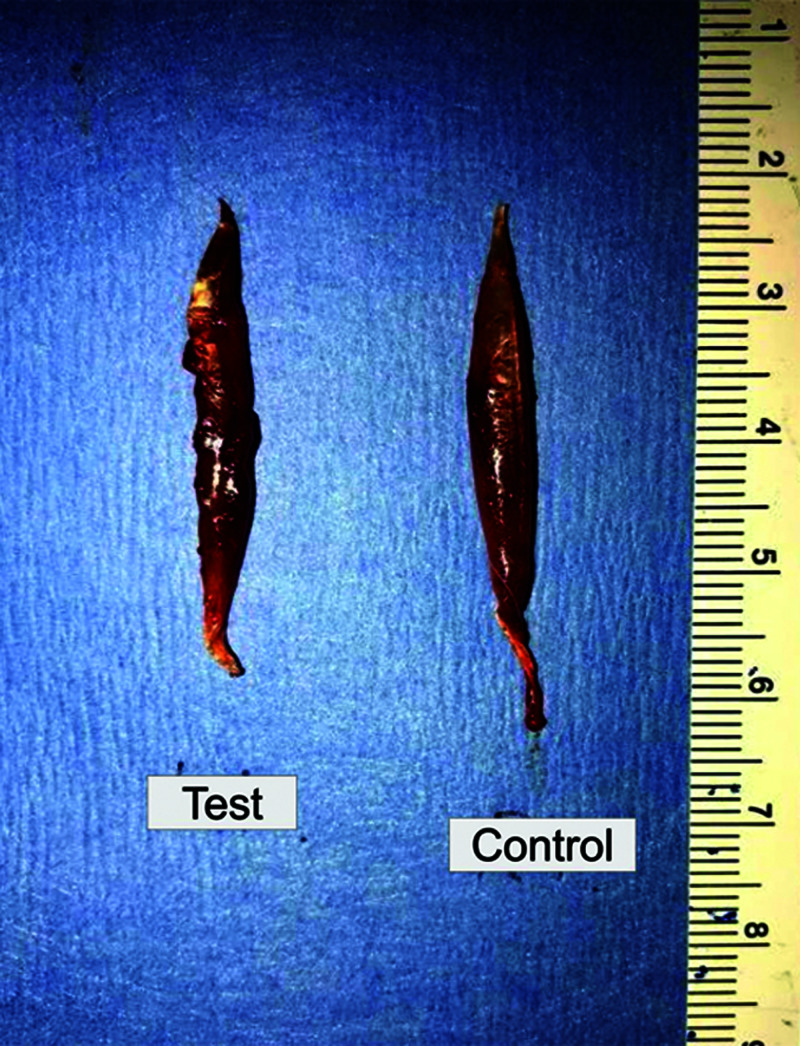
EDL Muscle Harvest The resected EDL muscles on the test side and the control side. On gross inspection, the test side appears atrophied compared to the control side. EDL, extensor digitorum longus

Bilateral EDL muscles were harvested and weighed (Figure 6).

Nerve biopsy

Nerve samples were obtained from the sciatic nerve proximal to repair and from peroneal nerve distal to the repair. These were fixed in 10% formalin, sectioned at 6 µm, and stained with Luxol fast blue with hematoxylin and eosin. Stained samples were imaged on an Olympus IX71 inverted microscope with an Olympus DP74 color camera and then examined to look for fascicles distal to the nerve repair.

Statistical analysis

Mean and standard deviation were calculated. EMG data were compared using a linear mixed model analysis (with Bonferroni post-hoc tests) or one-way analysis of variance (ANOVA) (with Holm-Sidak post-hoc tests) as indicated. Cohen's d values (equal group size between groups) or Hedges' g values (unequal sample size between groups) were calculated as a measure of effect size (socscistatistics.com/effectsize/default3.aspx) [9], where values of 0.2, 0.5, and 0.8 correspond to small, medium, and large effect sizes, respectively. The level of statistical significance (α) was set to 0.10 for all analyses of this pilot study.

## Results

Attrition

One rat in ETE died three months after surgery with unknown causes and we were not able to collect any data from this rat. Two rats in ETE and one rat in HT group died during the second surgery. We were able to collect the weight of EDL and nerve biopsy from these rats.

Threshold of stimulation

For the ETE group, the mean threshold of stimulation was 183.3 ± 55.0 µA for the test side and 213.6 ± 128.0 µA for the control side. For the HT group, the mean threshold was 3707.5 ± 1837.2 µA for the test side and 748.7 ± 501 µA for the control side. For the MHWT group, the mean threshold was 656.6 ± 414.3 µA for the test side and 851.6 ± 337.7 µA for the control side (Table 1).

**Table 1:** EMG results: mean values ± standard deviation and total number for each test. *Indicates p < 0.10 compared to other groups. ETE, end-to-end; HT, helicoid technique; MHWT, modified helicoid weave technique; TFC, tetanic force of contraction; EDL, extensor digitorum longus

	ETE		HT		MHWT	
	Test	Control	Test	Control	Test	Control
Threshold of stimulation (µA)	183.3 ± 55.0 (n = 3)	213.6 ± 128.0 (n = 3)	3707.5 ± 1837.2 (n = 4)	748.75 ± 501 (n = 4)	656.66 ± 414.3 (n = 6)	851.66 ± 337.7 (n = 6)
Threshold ratio	0.98 ± 0.32 (n = 3)		11.81 ± 11.78 (n = 3)*		1.04 ± 0.99 (n = 6)	
TFC (mg)	23.33 ± 11.54 (n = 3)	32 ± 0 (n = 3)	23 ± 3 (n = 3)	34.4 ± 5.54 (n = 5)	14 ± 4.56 (n = 6)	40.8 ± 2.28 (n = 5)
Wet EDL weight (mg)	139.6 ± 25.2 (n = 5)	185.6 ± 14.4 (n = 5)	123.5 ± 14.9 (n = 6)	174.8 ± 14.9 (n = 6)	120.1 ± 24.5 (n = 6)	20.1 ± 22.6 (n = 6)

Since the threshold of stimulation depends on various factors including temperature, pH, and anesthesia, we used the threshold ratio as a between-groups comparative measure. Threshold ratio was defined as threshold on the test side divided by threshold on the control side. Test and control measurements were taken simultaneously to eliminate the confounding factors. The mean of threshold ratio were 0.98 ± 0.32 for ETE (n = 3), 11.81 ± 11.78 for HT (n = 6), and 1.04 ± 0.99 for MWHT (n = 3). We compared the threshold ratios using one-way ANOVA. A significant main effect was found at the pilot study significance level of p < 0.10 (F(2,9) = 4.171; p = 0.052); Holm-Sidak post-hoc analyses revealed a significant difference between HT and both MHWT (p = 0.069) and ETE (p = 0.082).

Nerve conduction

**Figure 7:**
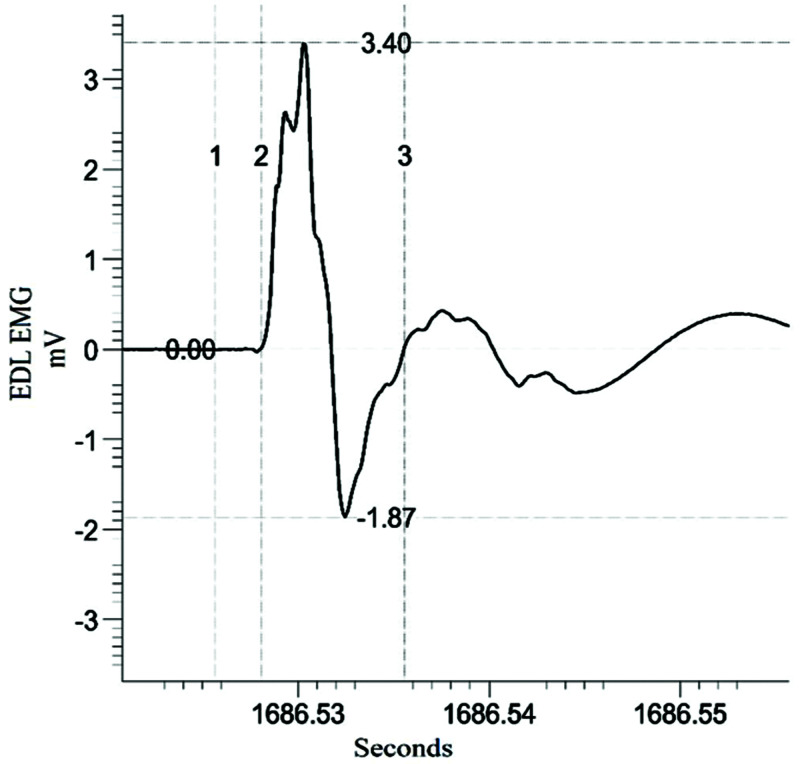
Example Electrical Stimulation (1 mA) and the Resulting M-Wave in the EDL Muscle Cursor 1 is placed on the stimulus marker and cursor 2 is placed at the beginning of the M-wave; the time between the two markers is the latency to respond. Cursor 3 is placed at the end of the M-wave when it intersects the midline; the time between cursors 2 and 3 is the duration of the signal. Horizontal cursors denote the amplitude of the signal, which could not be measured in all traces due to clipping. EDL, extensor digitorum longus

Linear mixed model analysis shows a significant main effect of repair group (F(2, 41) = 3.829; p = 0.03) and injured versus uninjured sides (F(1, 54) = 19.808; p < 0.001) on the latency to respond to stimulation. Due to small sample sizes per group, the inherently conservative Bonferroni post-hoc analysis did not reveal any significant differences among the groups. A significant main effect of injured versus uninjured sides (F(1, 81) = 10.117; p = 0.002) was found for the duration of the response but suffers from the lack of post-hoc significance (Figure 7, Table 2).

**Table 2:** Results of EMG latency to response and the duration of response in the EDL muscle No significant differences were detected between intensities and therefore the data have been collapsed into overall means with SD. Latency: significant main effects of side (a vs. b; p < 0.001) and significant main effect of group (a, b, c vs. a, b, c; p = 0.03). Duration: significant main effect of side (a vs. b; p = 0.002). Post-hoc comparisons were not significant due to low sample size and conservative nature of linear mixed model post-hoc comparisons. SD, standard deviation; ETE, end-to-end; HT, helicoid technique; MHWT, modified helicoid weave technique; EMG, electromyography; EDL, extensor digitorum longus

Side tested	Group	Latency (ms), mean (SD)	Duration (ms), mean (SD)
Uninjured	ETE	1.55 (0.32) a, a	6.52 (2.26) a
	HT	2.06 (0.65) a, b	5.67 (2.03) a
	MHWT	2.17 (0.48) a, c	7.23 (2.11) a
Injured	ETE	2.96 (0.90) b, a	4.91 (2.63) b
	HT	1.94 (0.80) b, b	3.94 (2.13) b
	MHWT	3.16 (1.21) b, c	5.51 (2.09) b

Taken together, these results show that latency and duration of response are useful outcome measures of this type of repair study.

Tetanic force contraction

The mean (±standard deviation) TFC in the ETE group was 23.3 (±11.54) mg for the test side and (32 ± 0) mg for the control side. For HT group, the mean (±standard deviation) TFC was 23 (±3) mg for the test side and 34.4 (±5.54) mg for the control side. For the MHWT group, the mean (±standard deviation) TFC was 14 (±4.5) mg for the test side and 40.8 (±2.28) mg for the control side (Table 1). A one-way ANOVA revealed no significant main effect between groups (F(2,9) = 2.921; p = 0.105). Since group sample sizes differed for this experiment, we calculated the Hedges' g value to determine the effect size. Compared to MHWT, both ETE (1.28) and HT (2.16) exhibited a large effect size; ETE versus HT exhibited a very low effect size (0.04).

Wet weight of EDL

The mean (±standard deviation) weight of EDL in the ETE group was 139.6 (±25.2) mg for the test side and 185.6 (±14.4) mg for the control side. In the HT group, mean (±standard deviation) weight of EDL was 123.0 (±14.9) mg for the test side and 174.0 (±14.9) mg for the control side. In MHWT group, the mean (±standard deviation) EDL weight was 120.0 (±24.5) mg for the test side and 201.0 (±22.6) mg for the control side (Table 1). A one-way ANOVA revealed no significant main effect between groups (F(2,14) = 1.201; p = 0.330). Due to different sample sizes between groups, we calculated Hedges' g value to determine effect size. Compared to ETE, MHWT (0.78) and HT (0.80) exhibited a large effect size; MHWT versus HT exhibited a low effect size (0.16).

Nerve biopsy

**Figure 8:**
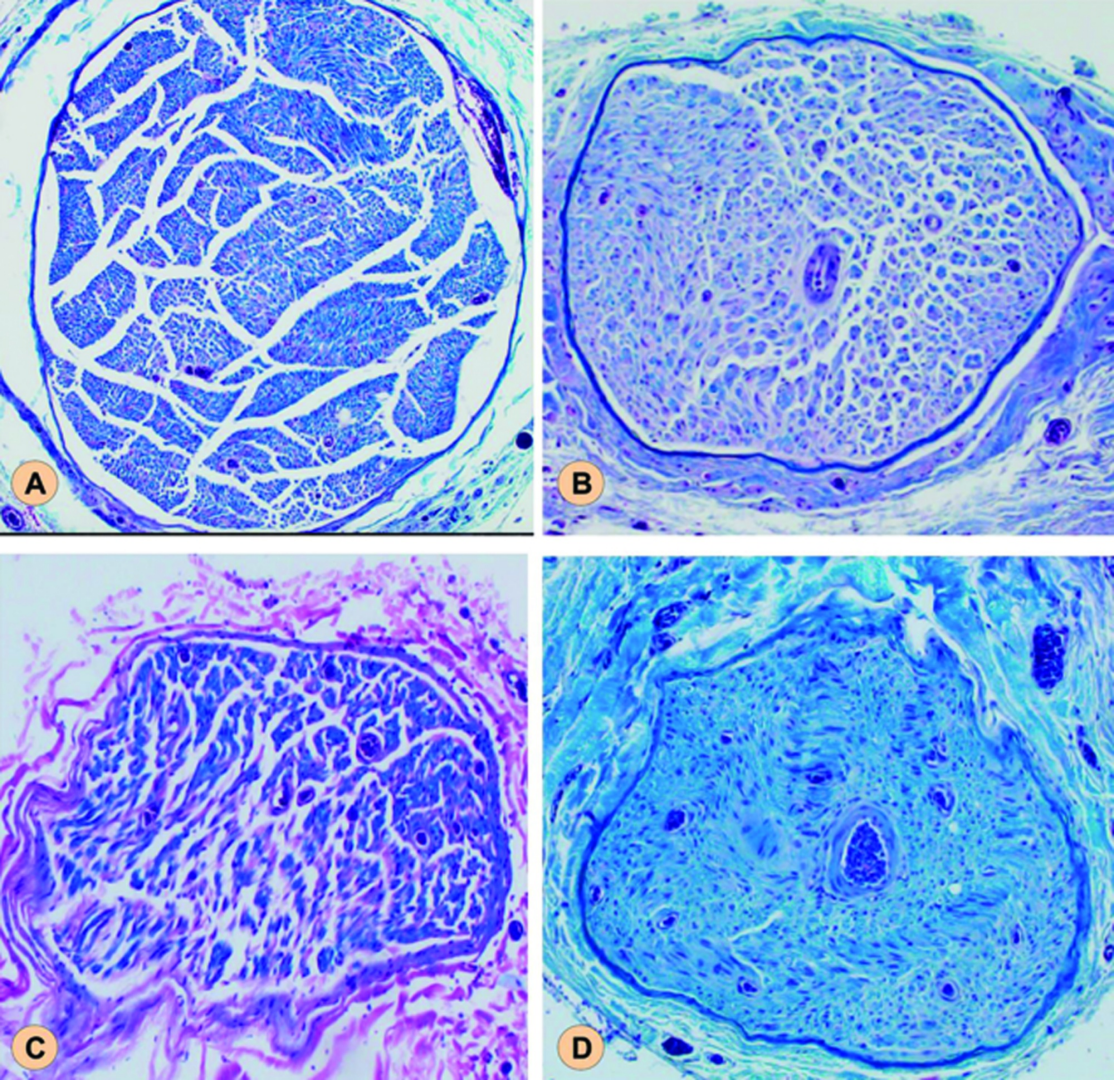
Nerve Fascicle Histology (A) Representative histological section from sciatic nerve proximal to repair. Section from peroneal nerve distal to the repair in (B) ETE group, (C) HT group, and (D) MHWT group. Nerve fascicles can be seen distally in three groups. ETE, end-to-end; HT, helicoid technique; MHWT, modified helicoid weave technique

Nerve biopsy was examined under the microscope. Fascicles were observed in the distal sections in all three groups. Due to the unsatisfactory quality of some of the slides, where fascicles were not cut perpendicular to their axis, we were not able to perform a reliable fascicular count (Figure 8).

## Discussion

The outcome of nerve grafting is multifactorial and depends on the type of nerve, duration since injury, site of injury, length of the graft, vascularity of the bed, and type of graft used. The effect of surgical technique on nerve grafting has not been the focus of most reports but should be a factor when considering outcomes. Nerve grafting is almost always performed by ETE technique. With the advent of nerve transfers, ETS repair has been increasingly studied [10]. In this technique, the distal limb of a transected nerve is reinnervated by coapting it into the side of an intact donor nerve. There have been various studies comparing ETS to ETE with varying results [11]. It is believed that ETS repair facilitates spontaneous collateral sprouting for sensory nerves; the more the perineurium is opened, the more spontaneous collateral sprouting will occur [12]. However, Hayashi et al. showed that for sprouting in motor nerves to occur, some form of axonal injury is needed [12].

This study was built on the work of Yan et al. [3-5] and shows successfully that nerve regeneration can take place across HT and MHWT. Theoretically, the HT is an improvement upon ETS repair. In traditional ETS repair, the end of the recipient nerve is placed on the side of the donor nerve by creating a perineural window. This can capture the collateral sprouting coming from that site, but sprouting events are location-specific and span across a limited area. Gordon et al. suggest that significant muscle function decline begins rapidly at 70% denervation [13]. Thus, it is imperative to direct a maximum number of nerve fascicles across the repair site, which the HT aims to achieve. The nerve graft is passed around the donor nerve across a broader area to capture not only more numbers but also more variety of donor axons. The HT provides a larger perineural window, enabling more nerve-to nerve interaction. Also, there is a larger area of controlled injury stimulating spontaneous collateral sprouting. By increasing the total area of contact, it is hypothesized that there will be an increased number and variety of axons that will cross over distally [3].

Yan et al. [3] compared HT to conventional ETS repair in a rat tibial-peroneal nerve model. The authors found that the mean potential amplitude, TFC of the EDL muscle, and moist weight of the EDL were found to be significantly better in the HT group. The authors also observed larger diameter fascicles on the HT side with denser, more mature nerve fibers. The mean number of regenerative nerve fibers distal to the repair site was higher in the HT group [3].

In a second study, the same authors compared HT to ETE in a rat model. In the first group, one end of saphenous nerve graft was attached to phrenic by HT and other end to MCN by ETE. In the second group, the phrenic was cut and the saphenous nerve graft was attached to both phrenic and MCN by ETE. The third group served as a control group with resection and ligation of MCN without any repair. The mean combined motor action potentials, TFC, wet muscle weight, and the mean number of regenerative myelinated nerve fibers distal to the repair site were statistically superior to in HT compared to ETE [4].

In a third study, Yan et al. compared HT to ETE in the vagus nerve to musculocutaneous transfer [5]. Similar to their second study, saphenous nerve grafts were obtained. In the first group, one end of the nerve graft was attached to the vagus in HT fashion and the other end to MCN in ETE fashion. In the second group, the nerve graft was attached ETE to phrenic and MCN. A third group served as control with ligation and division of MCN without any repair. The mean compound muscle action potential (CMAP), TFC, biceps muscle weights, and recovery ratios of regenerated nerve fibers were significantly better in the HT group than ETE and control.

Thus, in all three studies, Yan et al. consistently showed better results of HT over both ETS and ETE [2-4]. Based on these results, HT appears more effective than the current gold standard ETE technique for nerve repair. Hence, it is necessary to test whether the results demonstrated by Yan et al. are reproducible. Furthermore, the senior author of this paper (T.M.T.) proposed a novel technique that builds on HT called the modified helicoid weave technique (MHWT). HT increases the total surface area for nerve-to-nerve interaction and also allows access to wider topographical area on the donor nerve. MHWT serves to increase both of these factors by providing nerve-to-nerve interaction, not only at the surface but also at the core. MHWT serves to extrapolate the effects of HT in three dimensions. As shown in Figure 3, MWHT involves passing the nerve graft twice through the substance of the donor nerve. Care is taken to gently denude the epineurium of the graft where it passes through the donor nerve to increase nerve-to-nerve interaction.

There are many important findings in this study. First, we built on the studies conducted by Yan et al. and prove that HT and MHWT both function as a means of nerve repair in a rat sciatic nerve model. A potential application of HT and MHWT can be neuroma in continuity, which is a surgical challenge. The axons can be given an alternate pathway to reach their intended target, without potentially losing the existing neurons. Second, HT and MHWT are being compared to ETE in a mixed nerve model for the first time. Previous comparisons were in phrenic-musculocutaneous and vagus-musculocutaneous models, both of which were motor nerve models. Despite being a large mixed nerve, both HT and MHWT repairs of sciatic nerve injury innervated distal targets. Third, one of the proposed hypotheses of HT and MHWT was that these techniques would allow sprouting nerve fascicles to selectively enter its target. The nerve grafts in HT and MHWT are not aligned in any way. Nevertheless, HT and MHWT both showed some improvement, proving the hypothesis that the motor fascicles were able to successfully find their target despite a tortuous course. The fourth finding of our study was that neither HT nor MHWT was superior to ETE. We did find that the threshold ratio of injured to uninjured side was significantly worse in HT compared to both MHWT (p = 0.069) and ETE (p = 0.082) at the pilot study level of p < 0.10. Cohen's d or Hedges' g values were calculated where appropriate as a measure of effect size [9], where values of 0.2, 0.5, and 0.8 correspond to small, medium, and large effect sizes, respectively. Compared to HT, ETE showed a large effect size (1.30); similarly, MHWT showed a large effect size compared to HT (1.29). In all other measures, the mean values of ETE were superior compared to HT and MHWT. HT and MHWT are technically more demanding than conventional ETE repair. Furthermore, due to overlap at both ends, a graft that is longer than the defect is needed.

Finally, our study had several limitations. Attrition of four rats during the course of the study had an impact on the sample size. However, certain parameters could be obtained before death and were used for analysis. Similarly, muscle weight and nerve biopsy were obtained in all rats. Nonetheless, having a larger number of rats in each group would have been beneficial. The second drawback was the inability to perform fascicular count in all the slides. We have preserved the tissue blocks to look into this in a possible future study.

## Conclusions

While the previous work by a single group showed significant advantage of HT over ETE, we were not able to reproduce similar results. In this study, we demonstrated that HT and MHWT techniques were successful in reinnervating the distal muscle. However, we did not find any parameters in HT and MHWT being superior to ETE. While the mean values in ETE groups were superior, a statistical significance could not be achieved. Loss of four rats adversely affected our sample size. A study with larger sample size may show statistical significance.
